# Simulated data for census-scale entity resolution research without privacy restrictions: a large-scale dataset generated by individual-based modeling

**DOI:** 10.12688/gatesopenres.15418.2

**Published:** 2024-10-18

**Authors:** Beatrix Haddock, Alix Pletcher, Nathaniel Blair-Stahn, Os Keyes, Matt Kappel, Steve Bachmeier, Syl Lutze, James Albright, Alison Bowman, Caroline Kinuthia, Zeb Burke-Conte, Rajan Mudambi, Abraham Flaxman

**Affiliations:** 1 Institute for Health Metrics and Evaluation, University of Washington, Seattle, Washington, 98195, USA

**Keywords:** Entity resolution (ER), microsimulation

## Abstract

**Background:**

Entity resolution (ER) is the process of identifying and linking records that refer to the same real-world entity. ER is a fundamental challenge in data science, and a common barrier to ER research and development is that the data fields used for this fuzzy matching are personally identifiable information, such as name, address, and date of birth. The necessary restrictions on accessing and sharing these authentic data have slowed the work in developing, testing, and adopting new methods and software for ER. We recently released *pseudopeople*, a Python package that allows users to generate simulated datasets with configurable noise approaching the scale and complexity of the data on which large organizations and federal agencies, like the US Census Bureau regularly perform ER. With pseudopeople, researchers can develop new algorithms and software for ER of US population data without needing access to personal and confidential information.

**Methods:**

We created the simulated population data available for noising with pseudopeople using our Vivarium simulation platform. Our model simulates individuals and their families, households, and employment dynamics over time, which we observe through simulated censuses, surveys, and administrative data collection systems.

**Results:**

Our simulation process produced over 900 gigabytes of simulated censuses, surveys, and administrative data for pseudopeople, representing hundreds of millions of simulants. A sample simulated population of thousands of simulants is now openly available to all users of the pseudopeople package, and large-scale simulated populations of millions and hundreds of millions of simulants are also available by online request through GitHub. These simulated population data are structured for use by the pseudopeople package, which includes additional affordances to add various kinds of noise to the data to provide realistic, sharable challenges for ER researchers.

## Introduction

Entity resolution (ER) is a foundational element of data science and has emerged as a crucial research task in a variety of disciplines, from the social sciences to epidemiology to forensics ^1^. Put simply, ER is the process of linking the records corresponding to a single “entity” (e.g., an individual person) from one or multiple data sources when there is not a unique key on which to join them. In this context, an entity may be anything a row of data corresponds to, for example a person, household, business, or establishment. Record linkage of administrative data can enable the analysis of events across government services and systems ^2– 4^.

For researchers who work with large-scale, individual-level data, such as those working with the US Census Bureau, ER typically uses personally identifiable information (PII) such as name, address, date of birth, or government-issued identification numbers. Protecting PII is crucial to safeguarding individuals’ privacy, security, and personal well-being in an increasingly interconnected and data-driven world. As such, restrictions on access to these data have presented a barrier to developing and testing new methods and software for ER ^1^. Although it is possible for some research to proceed using perturbed or synthetic data or for researchers to work with confidential data in a secure data enclave, it seems that technical barriers inherent in these approaches have prevented them from overcoming these barriers at present.

In 2021, the US Census Bureau (USCB) awarded a cooperative agreement to the University of Washington’s Institute for Health Metrics and Evaluation (IHME) Simulation Science team to expand and improve ER methodological research and technology ^5^. As part of this work, we have used simulation to address the research barrier caused by PII. Through the development of a simulated version of various administrative datasets, including a simulation of the confidential data gathered by the USCB, we hope to help researchers develop new techniques for linking datasets together that are compatible with the privacy protections necessary for such sensitive and consequential information – and to do so without needing access to the real data. The goal of the generation of these data is to use them for ER methods research, and the datasets themselves are not intended to replicate or reconstruct protected data for social scientific research. It should be noted that our team is not the first to attempt such a data synthesis project. Prior approaches include the Australian National University’s “Freely extensible biomedical record linkage” ^6^ and Data Generator and Corruptor projects ^7^; and from the University of Arkansas Little Rock, the synthetic occupancy generator approach ^8^. There is also relevant work from the University of Edinburgh, which developed an R package for producing synthetic data called synthpop ^9^; and from the United Kingdom Ministry of Justice, which developed synthetic data for testing the Python package Splink ^10^. Recently another group has use simulation specifically to generate data for record linkage, which might be considered the first paper to use microsimulation for generating data for record linkage ^11^. There are certain features of our project, however, that differentiate it from other efforts, most notably the scale of our simulated data: We have simulated a 100% sample of the USA over 20 years.

We have recently released pseudopeople, a Python software package that allows users to generate realistic simulated data about a fictional United States population over multiple decades. Both the generation and distribution of the dataset are governed by a system of “relational governance” ^12^, in which data subjects play a central role; a paper on our governance approach is currently under preparation ^12^. The package produces simulated datasets similar to census, survey, and administrative datasets routinely used by USCB in their data linkage practice, and it allows the user to configure the levels of noise in each dataset. This includes noise that leaves fields blank, chooses wrong options, replaces names with nicknames and fake names, swaps months and days in dates, misreports ages, and writes wrong digits in numbers and zip codes, as well as adding phonetic, optical character recognition, and typographical errors, following the approach pioneered by Christen and colleagues ^6, 7, 13^. Readers interested in more details on how our pseudopeople software adds noise to the data generated by this simulation are referred to the pseudopeople documentation website, pseudopeople.readthedocs.io, which includes implementations and extensions of many of the data corruption approaches listed in the previous paragraph.

The simulated datasets generated by pseudopeople are based on the results of an individual-based microsimulation built with our Vivarium simulation framework. This simulation is calibrated with real, publicly accessible data about the United States population, including realistic household and family structures, at a large scale. The purpose of this data note is to describe this simulation, which we hope will aid researchers in using our pseudopeople package to develop new algorithms and software. 

## Methods

### Using Vivarium

Vivarium is a mature, open-source simulation framework ^14^ that uses standard scientific Python tools, such as NumPy and pandas ^15, 16^. A simulation in Vivarium consists of user-written components that encapsulate the simulation logic, a machine-readable model specification that describes what components are in the model and how they are configured, and a data file containing all data used in the simulation. The framework provides a set of services to assist users in writing their model components, an engine for executing the simulations from both an interactive Python session and from the command line, and abstractions to help manage and format model input data. Models built in Vivarium are typically individual-based, representing people in a population as agents or “simulants,” each with their own age, sex, and other characteristics relevant to the specific model. They typically use discrete “time steps” at which events may occur. In this work, each Vivarium simulant represented a person living in the United States, and each discrete time step included changes relevant to data used in record linkage, such as births, deaths, moving to another address, and changing jobs.

We use a pre-established workflow when developing a Vivarium simulation, with roles for researchers and software engineers. The researchers lead the model development process through background research, conceptualizing modeling strategies, validating strategies with domain experts, guiding the conceptual development of the modeling software, and generating analytics for simulation inputs and outputs. The software engineers lead the development of simulation code, including model components and outputs, and tools supporting model and input data analytics.

In the following sections, we will cover the different input data sources and data processing strategies used to inform our simulation of the US population. We describe the Vivarium model components for simulant characteristics including basic demographics, household structure, mortality, fertility, migration, and employment dynamics. We also describe the addition of simulant names, physical addresses, employer names, and other attributes which we implemented as a post-processing step, rather than during the simulation itself. 

### Simulation time

We initialized our simulation to begin on January 1, 2019, and step forward in time with 28-day time steps until the simulation clock exceeded May 1, 2041. We chose this time step duration to balance the complexity of changes in demographics, housing, employment, etc. with the computational demand of running a simulation with over 300 million simulants.

### Concept model

**Figure 1.**
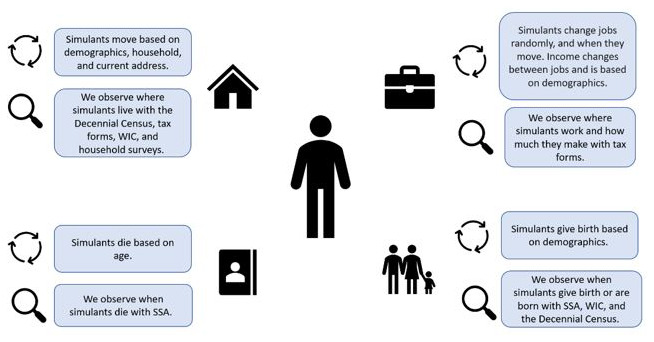
Simplified version of simulation concept model diagram, denoting the four different overarching simulation events that can occur (migration, employment, mortality, and fertility) and how each of these events are observed during the simulation run.

**Figure 2.**
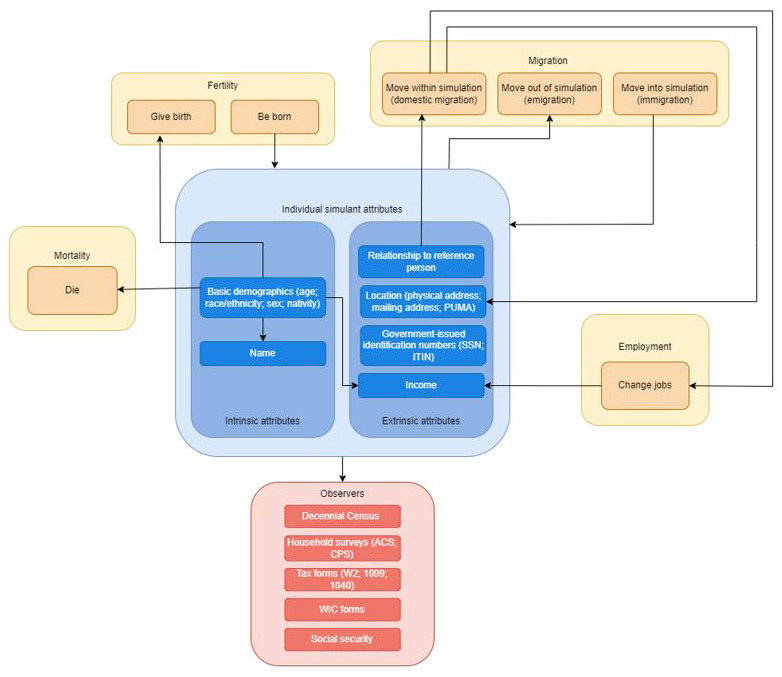
Concept model diagram showing the interaction between different components of the simulation, illustrated at the level of an individual simulant. Each arrow in this diagram represents a dependence between two distinct components of the simulation; an arrow from component X to component Y indicates that X affects Y (for example, the employment component simulates changes in jobs, which leads to a change in income; the basic demographics of a simulant affect the probability of death so that older simulants are realistically more likely to die during a simulation time step).

The concept model diagrams ( Figure 1 and Figure 2) provide a visualization of the logical dynamics underlying this simulation and indicate how the various components of the simulation relate. The simulation components can be divided into three overarching categories:

i) simulation events (i.e., birth, death, migration, and employment change),
ii) simulant attributes (i.e., demographics, household structure, location, and government-issued identification numbers such as SSNs), and
iii) simulated dataset observers (i.e., how the simulants are observed over the course of the simulation, through routinely collected surveys and administrative datasets, such as the Decennial Census, tax forms, household surveys, and government-related social safety programs).

Figure 1 shows what influences the occurrence of each simulation event and how these events are captured in our data collection, while Figure 2 shows how the simulant components interact with one another at the individual level. When a simulant undergoes an event (e.g., gives birth, changes jobs, changes address), the simulant’s attributes change accordingly. Those attributes are then captured by the observers.

***Input data.*** We informed the simulated datasets we developed for pseudopeople using open-source input data, including data released publicly by the Social Security Administration (SSA) and the USCB. We informed physical addresses from the training data of the Python package libpostal, as repackaged by the deepparse project ^17^. In the sections that follow, we elaborate on how we used these data sources, and how our simulation could be extended to be even more realistic in future work.

***Basic demographics.*** We initialized the simulated population’s demographic characteristics, including age and date of birth, race/ethnicity, sex, nativity (i.e., whether a simulant was born within the US), geographic area, and household structure by sampling from the 2016–2020 ACS Public Use Microdata Sample (PUMS) ^18^. By sampling from PUMS, we were able to match the univariate distribution of each attribute as well as joint distributions of arbitrary complexity between the attributes at the Public Use Microdata Area level, while also preserving structure within sampled household units. For instance, the PUMS data capture the age distribution of people in America, where more people were born from 1945 to 1965 than from 1965 to 1985. The PUMS data are not without limitations, however. For example, the granularity of the PUMS data is limited by privacy considerations, and specific details that might be crucial for a detailed analysis are sometimes obscured, which could affect the precision of simulations based on these data, especially in socioeconomic and health-related contexts.

Age is reported in PUMS in floored integer years, but our simulation uses precise ages in fractional years. We assigned simulants a uniformly random precise age consistent with their nominal age as sampled from PUMS. For ER research and development, it was particularly important that we did not generate simulants who are much more similar to one another than would be expected in a real population, which would make linkage unrealistically difficult. Our simulated population is the size of the US population, but every simulant is initialized from a person in PUMS, which is a 5% sample of the US. Therefore, many simulants are created from the same person in PUMS, which could create unrealistic clustering. To decrease similarity without assuming total independence between attributes, we perturbed age values at sampling time. In different components of the simulation, we sampled different entity types from the PUMS: entire households, individuals living in group quarters (GQs), or individuals living in households (non-GQs). For each entity sampled, we added a random age shift taken from a standard normal distribution to that entity’s age value(s). When perturbation led to a negative age value, we flipped the negative age value’s sign. We then defined each simulant’s date of birth to be consistent with their precise age.

Sex is reported in PUMS as binary (male or female), so we initialized a sex attribute this way as well for each simulant. We mapped separate PUMS indicators of race and ethnicity to a single composite “race/ethnicity” indicator, with the following exhaustive and mutually exclusive categories: “White,” “Black,” “Latino,” “American Indian and Alaskan Native,” “Asian,” “Native Hawaiian and Other Pacific Islander,” and “Multiracial or Some Other Race.” We defined these categories in accordance with the guidelines provided by the US Office of Management and Budget (OMB) ^19^. Nativity describes whether a simulant was born in the United States or elsewhere, and we modeled this as a binary variable in our simulation. We used this nativity attribute to inform the likelihood that the simulant had a Social Security Number (SSN). Table 1 provides a sample of the basic demographics present in the simulated population used in pseudopeople (note that these are entirely simulated data and therefore do *not* constitute Confidential Unclassified Information under US Code).

**Table 1.** Sample of basic demographics from simulated population. These are entirely simulated data and therefore do *not* constitute Confidential Unclassified Information under US Code.

Simulant ID	First name	Middle initial	Last name	Age	Date of birth	Sex	Race/ethnicity
2	Melanie	L	Herrod	26	8/5/1993	F	White
3	Jordan	C	Herrod	26	12/20/1993	M	White
923	John	E	Mckeever	77	6/29/1942	M	Black
6176	Gail	K	Durand	67	1/3/1953	F	Multiracial or Other
18770	Ann	J	Molina	60	10/24/1959	F	Latino

***Household structure.*** Our simulants lived in either residential households or group quarters (GQ). We used the ACS PUMS data to inform the residential household structure regarding how each simulant is related to a reference person in their household. Simulants living in GQ do not have such a relationship and GQs do not have a reference person. Residential households and GQs have geographic locations as well as physical and mailing street addresses, which may be different, because some residential households receive mail at a PO box (we do not simulate other kinds of mailing-only addresses, such as rural route addresses).

PUMS data were not sufficient to identify precisely which type of GQ each simulant resided in; they only provided information on whether it was an institutional or non-institutional GQ. We subdivided institutional GQ into three mutually exclusive and collectively exhaustive categories of carceral, nursing homes, and other institutional. We also subdivided non-institutional GQ into college, military, and other non-institutional. We chose a GQ type uniformly at random for each simulant out of the three types consistent with their institutional/non-institutional status.

For simulants living in residential households, we modeled a relationship to the reference person of their household based on the relationship values in the PUMS ^20^. Possible relationship values were reference person, biological child, adopted child, stepchild, sibling, parent, grandchild, parent-in-law, child-in-law, other relative, roommate, foster child, other non-relative.

***Mortality.*** To model mortality, we used our standard Vivarium approach, informed by data from the age- and sex-specific estimate of all-cause mortality for the US in 2019 as produced by the IHME Global Burden of Disease Study ^21^. When a simulant who was the reference person in a non-GQ household died, we made the oldest remaining simulant in that household the new reference person and updated all other relationships (this produces some households with an unrealistically young simulant as the reference person). Unlike many of our past Vivarium simulations, we did not model the underlying cause for any simulant’s death. However, we could extend this simulation to model specific causes of death in future iterations of the simulation, such as to facilitate research and development in cancer registry linkage applications.

***Fertility.*** We used our standard Vivarium approach to an age-specific fertility model in which each female simulant has a probability of having a birth event at each time step, derived from the age-specific fertility rate for the USA. In the current version of our model, only one female parent is identified, representing the simulant who gave birth. The birth event is considered to occur at a randomly chosen time during the 28-day time step, which informs the date of birth and age of the simulants born. We select a random 4% of birth events to be the birth of twins (two newborn simulants), and for the other birth events we add a single newborn simulant. We expect that the inclusion of twins will create some particularly challenging ER data, where simulants have the same last name, address, and date of birth. We do not include adoption or any other complexities of family structure.

The newborn simulant inherits certain attributes from their mother simulant, including household, race/ethnicity, and last name (recall that the simulation associates a newborn with only a single parent, so these attributes are inherited from this individual unambiguously). These simplifying assumptions allowed us to avoid modeling the complex dynamics of relationships but precluded us from following the dominant patriarchal naming pattern present in the US. The nativity of children born in the simulation is set to reflect that they were born in the US; therefore, all children born in the simulation are assigned an SSN. Additionally, we assigned newborns a relationship to the reference person in their household (which is also their parent’s household) based on the relationship between their parent and the household reference person, using a set of logical business rules.

***Migration.*** We attempted to include accurate patterns of migration in our simulation, as migration leads to changing addresses, which constitutes an important challenge in ER. As with basic demographics, all data informing migration in our simulation come from ACS PUMS. We used PUMS to calculate migration by demographics. There are a huge number of attributes that could explain moving behavior, and they may interact in complex ways in the real world. We modeled only some of this complexity and captured three types of household and individual migration events: migration within the simulation (domestic migration), migration into the simulation (in-migration), and migration out of the simulation (out-migration).

**Domestic migration**

We modeled domestic migration events as happening at a rate determined by age, sex, and race/ethnicity; we held these rates constant across time in the simulation. Individual domestic migration caused a single simulant to move and might reflect an individual moving out of their current living situation (i.e., GQ or residential household) and establishing a new one-person household, moving into GQ, or joining an existing residential household as a non-reference person. For individual migrations in which a simulant establishes a new household, we always classified the simulant as the reference person. For individual migrations in which a simulant joins an existing household, the simulant is always classified as an “Other nonrelative.” We assumed that simulants have at most a single individual migration event per time step.

When a simulant who was the reference person in a non-GQ household moved, we assigned the oldest remaining simulant in their household to be the reference person and updated all other relationships in the household according to logical business rules.

Household domestic migration caused an entire household of more than one simulant to move as a unit. As with individual migration, we calculated the rate of household migration per household-year and stratified by demographics. Because households do not have overall demographic characteristics, we used the demographics of the reference person for this stratification. Unlike individual migration, we did not change any relationships in household migrations.

We used a simplifying assumption that all simulants who moved and were of working age (which we define as age 18 and older) changed employment.

**International immigration**

We modeled immigration by adding new simulants to our simulation to represent individuals moving into the US from other countries. We sampled simulants immigrating to the US from the subset of the 2016–2020 ACS PUMS who had immigrated to the US in the year before they were surveyed and did not perturb their age. This approach relies on our assumption that the number-per-year and demographic characteristics of recent immigrants in the 2016–2020 ACS PUMS will not change substantially for all future years of the simulation.

We modeled three kinds of immigration events in our simulation: household moves, GQ person moves, and non-reference-person moves. As with domestic migration, a household move is when an entire non-GQ household enters from outside the country as a unit, preserving relationships within the unit. A GQ person move is when a simulant enters from outside the country and joins group quarters. Because simulants who reside in GQs do not have tracked relationships in PUMS or our simulation, these moves have no relationship structure. Lastly, a non-reference-person move is when an individual simulant enters from outside the country and joins an existing non-GQ household with some relationship other than “reference person.”

We used the weighted number of last-year immigration events of each type from the ACS PUMS to inform the yearly rate at which immigration events of each move type occurred in our simulation. We simulated constant rates over time and did not model seasonal or temporal fluctuation in immigration. 

**International emigration**

Emigration occurs when a simulant leaves the US to live in another country. We used the Net International Migration (NIM) estimates from the Census Bureau’s Population Housing Unit (PopEst) program to determine the number of emigrants per year, by subtracting immigration numbers from ACS to isolate emigration. The NIM estimates are made by the PopEst team by combining information about immigration from ACS with information about emigration from demographic analysis (for those born outside the US) and analysis of foreign censuses (for those born in the US) ^22^.

There are three types of emigration events that can occur in our simulation: household moves, GQ-person moves, and non-reference-person moves. These cause an entire household, a GQ person, or a household member who is not a reference person to leave the US, respectively. We stratified emigration rates by age group, sex, race/ethnicity, nativity, and US state of residence, and we assumed that these stratified rates were constant over time, without a long-term trend or seasonal variation. We stopped tracking households and individuals after an emigration event and assumed that they would not return to the US or appear in any pseudopeople data after they had emigrated.

***Employment dynamics.*** We consider all simulants aged 18 years or older to be working age; all such simulants either have an employer or are considered unemployed. We only allow a single employer at a time for each simulant. We initialized the working-age simulants to be unemployed, employed in the military, or employed otherwise, and we considered the military to be a single employer. To employ the rest of the simulants (those with non-military jobs), we generated employers with an initial size attribute chosen from a skewed distribution to ensure that there are a few large employers and many small employers. In order to assign individual simulants to employers such that the size attribute is (roughly) accurate at the population level, we selected each simulant’s employer from the categorical distribution where the probability of each employer is proportional to its initial size attribute.

Working-age simulants (including those who are unemployed) change employment randomly at a rate of 50 changes per 100 person-years (a rate we selected subjectively to provide an appropriate challenge in record linkage). When a simulant changes employment, we sample a new employer with the same procedure used at initialization. This approach to selecting a new employer ensures that at the population level, the number of simulants employed by any given employer will remain roughly proportional to the initial size attribute sampled for that employer.

We also simulate income, which affected which datasets a simulant appeared in. For instance, the WIC dataset only recorded simulants with household income below a certain threshold. We approximated the income distribution with a log-normal distribution for each age group, sex, and race/ethnicity combination, fit to the ACS PUMS. See Appendix 1 for more detail on the distribution parameters used for each demographic group. We simulated only income earned through wages; unemployed simulants had no income. To simplify our model, we assumed statistical independence between wages and employer for employed simulants.

### Post-processing

We added some elements to the simulated data after the simulation ran. This includes features that would require additional computing resources to track for the simulation’s duration, such as simulant names (first and last), employer names, and government-issued identification numbers (i.e., SSNs and ITINs).

We developed simulant first and last names based on two distinct data sources: first and middle names are sourced from SSA data, which allowed use to match name frequencies to age and sex; while last names are generated from Census data with hyphens and spaces added to make linking tasks realistically more challenging, which allowed use to match the frequencies by race/ethnicity ^23, 24^. We generated SSNs in accordance with the current algorithm used to issue unique SSNs. We selected the first three digits uniformly at random from 001 to 899, excluding 666; the next two digits from 01 to 99; and the final four digits from 0001 to 9999 ^25^. We generated Individual Taxpayer Identification Numbers (ITINs) for simulated 1040 filings by simulants without an SSN using a similar process ^26^.

We based our simulated employer names on a database of 5,321,506 “location names” from the SafeGraph “Core Places of Interest USA” dataset released in June 2020 ^27^. To create a representation of bigrams from this dataset, we constructed a directed multigraph. Each word in a location name was treated as a node, and we included special <start> and <end> nodes. We included a directed multi-edge for each occurrence of a word pair in sequence in each location name. To generate simulated employer names, we performed a random walk through the bigram graph. Starting from the <start> node, we traversed directed edges selected uniformly at random until we reached the <end> node or exceeded a predetermined maximum path length. We then combined the words associated with each node that was encountered along the path to form the simulated employer name. This approach resulted in a diverse range of names that maintained a realistic quality. In the sample data included with the pseudopeople package, the W2 and 1099 employer names in 2020 include 212 distinct names and the three most commons are “San Benito Martinez Landscape Supply”, “Tony's Family Practice Inc”, and “Pikes Creek Campground”.

## Dataset validation

For dataset validation, we followed the standard workflow used across all of our Vivarium models, using a process often referred to as verification and validation (V&V) ^28^. In this process, model results are verified by the research team by checking that a given model approximately replicates target values it was explicitly designed to replicate (e.g., verifying that the proportion of simulants living in group quarters as opposed to individual households matched the value specified by the research team). Results are also validated by the research team, ensuring that model results are logically viable or sensible (e.g., checking that the US population size and structure does not change drastically over the time period modeled). In the event that model results did not meet verification and validation criteria, model implementation and/or design were iteratively adjusted appropriately until criteria were satisfied.

We validated pseudopeople datasets through automated testing conducted on the engineering side as well as manual, systematic testing of the simulated population and post-processing data on the research side. In an effort to be as systematic as possible with our user-led data testing processes, we aspired to specify our verification and validation strategies before the synthetic population model was developed by our engineers. For example, we used an interactive simulation in a Jupyter Notebook to verify that simulants were dying at the age- and sex-specific all-cause mortality rates estimated for the USA by the IHME Global Burden of Disease Study.

### Dataset limitations

There are a variety of limitations to our simulation strategy which may affect its ability to reflect real-world dynamics, including but not limited to those regarding migration, employment, physical or mailing address, guardianship, household structure and simulant relationships, and simulant identification.

For instance, to make possible the simulation of complex migration dynamics, there are a series of assumptions we made regarding how simulants and households move around, into, and out of the simulation. We assumed that domestic and international migration do not change over time, but rather remain at the average rate from 2016 to 2020 in each future year of the simulation. When a simulant moves, we assume that their mailing address, physical address, and employer all change. In addition, complexities particular to household sub-structure interacting with migration are largely not captured in our simulation. For instance, a child can move out of their household without a parent, or a simulant could move without their spouse into a different household. Additionally, we assume that relationship does not affect emigration rates and that all household types are equally likely to have a simulant move out of or into them. Furthermore, for any individual migration of a simulant from one household into another, we assign the relationship “other nonrelative” in their new household. Thus, as time passes within the simulation, the proportion of households with irregular relationship structures grows. In the sample data included with the pseudopeople package, the 2020 decennial census has 4% of rows have “Other nonrelative” as relationship to the reference person, and in 2030 this rises to 16%. Even in the early years of our simulation, it is possible that there are rare, but challenging, household structures which are not sampled in ACS and therefore not represented in our data either, for example a very small fraction of very large households might present a problem in real-work linkage work that would not be identified when testing with pseudopeople data.

Similarly, there are several assumptions that we made to simplify our model of employment dynamics in our simulation. We do not model retirement, and each simulant can only have one employer at a time. There is a myriad of business dynamics that we currently do not model, including new businesses opening, existing businesses closing, business name changes, or business mergers and acquisitions. As with household physical and mailing addresses, when a business address is vacated, it is not reused. In effect, this likely makes business record linkage with these data easier than it will be in practice.

Our age-specific fertility and mortality models do not account for variations related to income or race/ethnicity, and in future iterations of this work, we wish to address more complicated dynamics between the various elements of our simulation.

There are also limitations in simulant identification because of privacy protections in the name data we have used. The data on first names excludes names with fewer than five occurrences while the data on last names included only names with at least 100 occurrences. Furthermore, we did not model the correlation between first and last names explicitly. We hope to address these limitations in future refinements of our model.

## Results

Our simulation process produced over 900 gigabytes of simulated censuses, surveys, and administrative data for pseudopeople, representing hundreds of millions of simulants. A sample simulated population of thousands of simulants is now openly available to all users of the pseudopeople package, and large-scale simulated populations of millions and hundreds of millions of simulants are also available by online request through GitHub ( github.com/ihmeuw/pseudopeople/issues). These simulated population data are structured for use by the pseudopeople package, which includes additional affordances to add various kinds of noise to the data to provide realistic, sharable challenges for ER researchers.

Table 2 shows a sample of the simulated data that might be found in administrative sources on income tax (note that these are entirely simulated data and therefore do *not* constitute Confidential Unclassified Information under US Code).

**Table 2.** Sample of tax data from simulated population. These are entirely simulated data and therefore do *not* constitute Confidential Unclassified Information under US Code.

Simulant ID	First name	Last name	SSN	Employer	Wages	Tax Form
4	Eric	Alonso Tellez	584-16-0130	Pikes Creek Campground	$10,192	W2
5	Erin	Alonso Tellez	854-13-6295	Red’s Dairy Queen	$28,355	W2
5	Erin	Alonso Tellez	854-13-6295	Warrensburg	$18,243	W2
5621	Derick	Castillo	674-27-1745	Nashville City Properties	$7,704	W2
5623	Heather	Castillo	794-23-1522	Ecr Whipple Oliver Finley Shoe Sensation	$3,490	1099

## Conclusions

By generating population data with complexity and scale comparable to that of large organizations and federal agencies, like the US Census Bureau, we hope to circumvent the common data privacy– and access-related barriers to ER research and development. We intend for this data note to serve as a comprehensive guide for researchers contemplating the use of pseudopeople to develop and test their fresh theories, algorithms, and software systems.

## Acronyms

**Table:** 

Acronym	Full Form
**ACS**	American Community Survey
**CPS**	Current Population Survey
**ER**	Entity Resolution
**GQ**	Group Quarters
**IHME**	Institute for Health Metrics and Evaluation
**ITIN**	Individual Taxpayer Identification Number
**NIM**	Net International Migration
**OMB**	Office of Management and Budget
**PII**	Personally Identifiable Information
**PUMA**	Public Use Microdata Area
**PUMS**	Public Use Microdata Sample
**SSA**	Social Security Administration
**SSN**	Social Security Number
**USCB**	United States Census Bureau
**V&V**	Verification and Validation
**WIC**	Special Supplemental Nutrition Program for Women Infant and Children

## Data Availability

While a small amount of pseudopeople data is openly available as part of the Python package, access to the full datasets will require users to be both transparent and accountable to a committee of interested parties, including civil society organizations, privacy experts, and data subject representatives. To read more about how to access the full datasets associated with pseudopeople, please visit our website at https://www.pseudopeople.org/.

## Software availability

**Source code available from:** https://github.com/ihmeuw/vivarium_census_prl_synth_pop

**Archived source code at time of publication:** https://doi.org/10.5281/zenodo.10967291 ^29^.

**License:** BSD 3-Clause License.

The steps to reproduce and run the software are available at the site listed above. Please note that although this software is freely available, running the simulation and post-processing at full-USA scale is a resource-intensive process that requires substantial computational resources. Our approach used parallelization across 334 individual jobs, with each job accommodating an optimal population size estimated to be between 750,000 and 1.5 million individuals. For a run of 1 million individual simulants, we used approximately 55 gigabytes of memory and a runtime of 21.5 hours.
