# Supplementary material for: Simulated data for census-scale entity resolution research without privacy restrictions: a large-scale dataset generated by individual-based modeling
Source: Gates Open Res. 2024 Oct 18;8:36. Originally published 2024 May 3. [Version 2] doi: 10.12688/gatesopenres.15418.2 (PMC11518969; doi:10.12688/gatesopenres.15418.2)
Supplement: Peer review history

# Reviewer report: Reviewer response for version 2

**DOI:** 10.21956/gatesopenres.17690.r38295

**Published:** 2024-10-28

**Authors:** Rainer Schnell

**Affiliations:** 1 University of Duisburg-Essen, Duisburg, Germany

The paper has improved a lot.

1. It is now clearer that detailed corruption is done within "pseudopeople".
2. However, neither the intended workflow nor the relationship between the input generated here, pseudopeople and vivarium is evident from the paper alone.
3. Since this is a microsimulation, a reference to the standard textbooks (for example, Handbook of Microsimulation, Practical Microsimulation or Spatial Microsimulation (...)) seems to be required.
4. Since the aim of the paper seems to generate records for ER, the dependencies of the changes of QIDs due to the simulated processes are essential for an unbiased estimate of successful ER. For example, when does a name change to which variant, caused by marriage, divorce, free will or naturalization? I cannot find a description in the text, although these processes are essential to the problems of ER in practice. If these processes are implemented, they need parameters derived from empirical studies, which should be referenced.
5. The authors state: "There are certain features of our project, however, that differentiate it from other efforts, most notably the scale of our simulated data: We have simulated a 100% sample of the USA over 20 years." Population microsimulation is common; for example, the UK models and the German Mikrosim project cover a whole nation for 20 years or more (which hardly makes sense in microsimulation). Furthermore, there is a Canadian and a Swedish microsimulation of the population. So, the special feature is that it is a US model. Neither the population covering aspect, the duration, nor the ideas of the main mechanisms of the microsimulation are new.

Are sufficient details of methods and materials provided to allow replication by others?

No

Is the rationale for creating the dataset(s) clearly described?

Yes

Are the datasets clearly presented in a useable and accessible format?

Partly

Are the protocols appropriate and is the work technically sound?

Partly

Reviewer Expertise:

microsimulation, record linkage, PPRL, census operations

I confirm that I have read this submission and believe that I have an appropriate level of expertise to confirm that it is of an acceptable scientific standard, however I have significant reservations, as outlined above.

# Reviewer report: Reviewer response for version 2

**DOI:** 10.21956/gatesopenres.17690.r38294

**Published:** 2024-10-22

**Authors:** James D. Gaboardi

**Affiliations:** 1 Oak Ridge National Laboratory, Oak Ridge, USA

I have no further comments.

Are sufficient details of methods and materials provided to allow replication by others?

Yes

Is the rationale for creating the dataset(s) clearly described?

Yes

Are the datasets clearly presented in a useable and accessible format?

Yes

Are the protocols appropriate and is the work technically sound?

Yes

Reviewer Expertise:

GIScience, Spatial Optimization, Geocomputation, Research Software Science

I confirm that I have read this submission and believe that I have an appropriate level of expertise to confirm that it is of an acceptable scientific standard.

# Reviewer report: Reviewer response for version 1

**DOI:** 10.21956/gatesopenres.16769.r36776

**Published:** 2024-07-17

**Authors:** Rainer Schnell

**Affiliations:** 1 University of Duisburg-Essen, Duisburg, Germany

This is an interesting project and an interesting article.

 My main concerns relate to the assumptions in using the names. The names are from top-lists and therefore do not contain rare names. The entropy of the QIDs is therefore underestimated, and linkage quality will be overestimated,

 Furthermore, first names and last names are used independently and this will also underestimate the entropy in real data sets. Again, linkage quality will be biased.

 By inflating ACS data, the same problem will occur. For example, rare household compositions, such as very large households, will be rare or even non-existing in ACS. Exactly these households will cause problems in real-world linkages. For example, similar names and birthdays at the same address are more in census data than in samples of 1 or even 5 % of the population. Furthermore, using name distributions independent of very large household compositions will cover up the main census linkage problem in my experience: Separating very similar persons. These "rare" cases of about very few percent of a population will cause underestimation of subpopulations using a biased sample (given the aim are census estimates).

 The terminology is a bit strange. For example, naming the simulated entities "simulants" is unusual in microsimulation (see, for example, O'Donoghue).

 Another example is “shards”, used a synonym for computational jobs. However, in general, a shard is

 is a horizontal partition of data in a database or search engine (cited from Wikipedia), not a job. There are many more issues like these, and it might be helpful to check the usage of the terminology again.

 I miss a clear statement, which parameters of the model are available within the programs provided or as parameter files. The time available for reviews do not permit running these programs for replication, therefore more much more details on what can be done with the data available at the GitHub link is required.

 If a corrupter for QIDs is part of the program collection is also unclear to me. If so, the details of the parameters and their dependency on the simulated processes are of prime interest. For example, the text states: "These simulated population data are structured for use by the pseudopeople package, which includes additional affordances to add various kinds of noise to the data to provide realistic, sharable challenges for ER researchers." Thefefore, is this paper describing "pseudopeople", "Vivarium", their joint usage or something else?

 Some previous work is not cited. For example, to the best of my knowledge, we have published the first paper on using microsimulation for generating data for record-linkage (Schnell/Weiand 2023).

 Furthermore, the book on linking techniques by Christen et al. discusses many previous generators and corrupters systematically.

Are sufficient details of methods and materials provided to allow replication by others?

No

Is the rationale for creating the dataset(s) clearly described?

Yes

Are the datasets clearly presented in a useable and accessible format?

Partly

Are the protocols appropriate and is the work technically sound?

Partly

Reviewer Expertise:

microsimulation, record linkage, PPRL, census operations

I confirm that I have read this submission and believe that I have an appropriate level of expertise to state that I do not consider it to be of an acceptable scientific standard, for reasons outlined above.

## References

[1] : Microsimulation of an educational attainment register to predict future record linkage quality. *Int J Popul Data Sci* .2023;8(1) : 10.23889/ijpds.v8i1.2122 2122 10.23889/ijpds.v8i1.2122 37649490 PMC10463005

[2] : Linking Sensitive Data.2020; 10.1007/978-3-030-59706-1 10.1007/978-3-030-59706-1

[3] : Practical Microsimulation Modelling.2021; 10.1093/oso/9780198852872.001.0001 10.1093/oso/9780198852872.001.0001

# Response

**Published:** 2024-10-02

**Authors:** Abraham Flaxman

**Affiliations:** University of Washington, Seattle, USA

This is an interesting project and an interesting article.

 My main concerns relate to the assumptions in using the names. The names are from top-lists and therefore do not contain rare names. The entropy of the QIDs [ADF1] is therefore underestimated, and linkage quality will be overestimated, and furthermore, first names and last names are used independently and this will also underestimate the entropy in real data sets. Again, linkage quality will be biased.

*Response: We appreciate the reviewer identifying these important limitations, and we have added text to our limitations section to call attention to them.  In future work, we hope to compare entropy from our approach and some of the datasets without such redactions, such as from voter registration data, and also to enhance our approach to have more realistic marginal distributions for first and last names as well as joint distributions over (first name, last name) pairs.*

 By inflating ACS data, the same problem will occur. For example, rare household compositions, such as very large households, will be rare or even non-existing in ACS. Exactly these households will cause problems in real-world linkages. For example, similar names and birthdays at the same address are more in census data than in samples of 1 or even 5 % of the population. Furthermore, using name distributions independent of very large household compositions will cover up the main census linkage problem in my experience: Separating very similar persons. These "rare" cases of about very few percent of a population will cause underestimation of subpopulations using a biased sample (given the aim are census estimates).

*Response: We have added to the limitations section to call attention to this limitation as well, and appreciate the reviewer highlighting it for us.*

 The terminology is a bit strange. For example, naming the simulated entities "simulants" is unusual in microsimulation (see, for example, O'Donoghue).

 Another example is “shards”, used a synonym for computational jobs. However, in general, a shard is a horizontal partition of data in a database or search engine (cited from Wikipedia), not a job. There are many more issues like these, and it might be helpful to check the usage of the terminology again.

*Response: This is due to some internal jargon that has developed over time in our multidisciplinary group at IHME, and we appreciate the reviewer identifying it.  We can easily can change our terminology to avoid some of the cognitive burden of unfamiliar language, and we have removed the term “shard” as part of this effort.  We appreciate the precision “simulant” brings to discussing data fields that would be considered confidential information if they were about a real person, however, and have kept this terminology, which we hope becomes more common over time.*

 I miss a clear statement, which parameters of the model are available within the programs provided or as parameter files. The time available for reviews do not permit running these programs for replication, therefore more much more details on what can be done with the data available at the GitHub link is required.

*Response: This can be an important limitation for users, and we have added details on the parameters which are inherent to the simulation and cannot be modified in the pseudopeople “post processing”.*

 If a corrupter for QIDs is part of the program collection is also unclear to me. If so, the details of the parameters and their dependency on the simulated processes are of prime interest. For example, the text states: "These simulated population data are structured for use by the pseudopeople package, which includes additional affordances to add various kinds of noise to the data to provide realistic, sharable challenges for ER researchers." Thefefore, is this paper describing "pseudopeople", "Vivarium", their joint usage or something else?

*Response: Our goal was to describe a specific simulation that we built with our Vivarium framework, to generate data for our pseudopeople package.  Upon reflection, this is sure to be really confusing for anyone outside of our team, and we apologize for not making it clearer!  We have added more detail about what is in pseudopeople and what is in this simulation; this was also confusing to reviewer 1.*

 Some previous work is not cited. For example, to the best of my knowledge, we have published the first paper on using microsimulation for generating data for record-linkage (Schnell/Weiand 2023).

*Response: Thank you for highlighting this relevant literature, to which we have added a citation.*

 Furthermore, the book on linking techniques by Christen et al. discusses many previous generators and corrupters systematically.

*Thank you for highlighting this, which is now even more important for us to refer to, since we have added information on the pseudopeople package that uses the simulated data described in the first draft of our paper, and the data corrupters included therein are substantially influenced by Christen et al’s FEBRL and GeCO methods.*

# Reviewer report: Reviewer response for version 1

**DOI:** 10.21956/gatesopenres.16769.r37051

**Published:** 2024-07-17

**Authors:** James D. Gaboardi

**Affiliations:** 1 Oak Ridge National Laboratory, Oak Ridge, USA

The authors present a thorough and holistic population simulation procedure with Vivarium & the means to access it with pseudopeople. This framework is operationalized through the use of publicly-available data products from the US Census Bureau, the Social Security Administration, and other auxiliary datasets. The overarching purpose of the framework is synthetic Entity Resolution (ER), and the implementation takes chronology and life events into account (e.g., age, residence change, birth, death). Person- & household-level records are simulated that are also linked to related demographic information, such employment and migration. The authors produce large-scale simulated census information for a span of 20 years into the future, a sample of which is entirely open-sourced while more detailed data is available through request.

 I am curious for the authors' insights into the potential of integration into other simulation frameworks, such as ChiSim ^1^ and UrbanPop ^2,3^. As a full disclosure, I am affiliated with the UrbanPop project.

  

1. C.M. Macal, N.T. Collier, J.Ozik, E.R. Tatara, and J.T. Murphy (2018). "Chisim: An agent-based simulation model of social interactions in a large urban area," in 2018 winter simulation conference (WSC), pp. 810820, IEEE. DOI: 10.1109/wsc.2018.8632409.
2. J.V. Tuccillo, R. Stewart, A. Rose, N. Trombley, J. Moehl, N.N. Nagle, and B. Bhaduri (2023) "UrbanPop: A spatial microsimulation framework for exploring demographic influences on human dynamics," Applied Geography, vol. 151, pp. 102844. DOI: 10.1016/j.apgeog.2022.102844.
3. J.V. Tuccillo and J.D. Gaboardi (2023) "Spatial Microsimulation and Activity Allocation in Python: An Update on the Likeness Toolkit," Proceedings of the 22nd Python in Science Conference, pp. 93-100. DOI: 10.25080/gerudo-f2bc6f59-00c.

Are sufficient details of methods and materials provided to allow replication by others?

Yes

Is the rationale for creating the dataset(s) clearly described?

Yes

Are the datasets clearly presented in a useable and accessible format?

Yes

Are the protocols appropriate and is the work technically sound?

Yes

Reviewer Expertise:

GIScience, Spatial Optimization, Geocomputation, Research Software Science

I confirm that I have read this submission and believe that I have an appropriate level of expertise to confirm that it is of an acceptable scientific standard.

## References

[1] An agent-based simulation model of social interactions in a large urban area. *winter simulation conference (WSC)* .2018;

[2] : UrbanPop: A spatial microsimulation framework for exploring demographic influences on human dynamics. *Applied Geography* .2023;151: 10.1016/j.apgeog.2022.102844 10.1016/j.apgeog.2022.102844

[3] Spatial Microsimulation and Activity Allocation in Python: An Update on the Likeness Toolkit. *Python in Science Conference* .2023;

# Response

**Published:** 2024-10-02

**Authors:** Abraham Flaxman

**Affiliations:** University of Washington, Seattle, USA

*Thank you for this positive assessment and fun opportunity to read up and think about; at a high level, it seems that some integration must be possible!  If I understand correctly, it appears that the time scale of the ChiSim and UrbanPop projects is substantially finer-grained than what we used in pseudopeople --- while they capture patterns of mobility over the course of a day, we have focused on internal migration patterns that might happen only once in a year.  That said, the general area is very similar, and the work in our setting might be useful for this line of research.  And vice versa!  Thank you for calling our attention to this work.*

# Reviewer report: Reviewer response for version 1

**DOI:** 10.21956/gatesopenres.16769.r36771

**Published:** 2024-07-17

**Authors:** Hye Chung Kum

**Affiliations:** 1 Population Informatics Lab, Texas A&M University, College Station, Texas, USA

This paper describes a python package that can simulate population data based on distributions of various individual and household characteristics in datasets released from the US census Bureau, with an explicit purpose for supporting and facilitating entity resolution (ER) algorithm and software development without accessing real confidential data. ER algorithms require access to personally identifiable information (PII) such as names, SSN, DOB making privacy a major concern in this type of research. Using simulated data, such as the one described in the paper is one approach to dealing with the privacy issues in ER. It relies on using the existing Vivarium simulation platform the same team developed, but attempts to incorporate some of the complexities of the ER problem with real data such as difficulties with twins.

 The paper is well written and easy to follow for the most part. Here are some questions/comments to consider for improvement.

 [Major comments]

- Data errors (e.g., typos in names and dob, flipped first/last name in different systems) or variations (e.g., nicknames, change in last name due to marriage, Jr and Sr) are a common source of difficulties in ER using real data which does not seem to be modeled yet. Maybe at least mention it as important future work.
- Similarly, duplicate records that exist in almost any real data make ER much more difficult because one cannot assume a one-to-one match unless the tables have been deduplicated first. One of the main difficulties in ER is that a duplicate record for one person cannot be mathematically differentiated from very similar records for 2 people such as twins, father/son, or even totally unrelated people (to date, I have met 2 people who told me after a ER talk that they know “of” this other person with the EXACT same first/last name and dob because they are in the same city and they have been confused before with the other person. Often it requires an explicit field stating that the person is a twin to be certain.
- I highly recommend reading the following paper for ideas for future work to make the simulation more realistic for ER research as well as adding a limitation section to the current paper. Such a section would be very important for potential users to know what the simulated data does not cover, and may need to still consider in their ER algorithm.
  - Goth G. Running on EMPI. Health information exchanges and the ONC keep trying to find the secret sauce of patient matching. Health data management. 2014;22(2):52-, 4, 6 passim.
- Also, consider the following for the limitation section. It seems that this simulated data may be more supportive of ER algorithms that leverage structure (e.g., household structure or based on clustering) without a pair generation. However, many real world applications do not have much structure information to use, and may need to still consider pairwise ER algorithms. Complexities for this type of ER algorithms may not have been well modeled here (see point above).
- A few sentences describing and talking about data governance/privacy concerns even for the simulated data in the introduction would be important. To my understanding, at a minimum a simulation of this scale may have simulatants that are too similar to real people, even if the data was all made up which may still give rise to privacy issues. It is very important that everyone becomes more educated on fundamental properties of information privacy. Such as, information privacy is not a binary property but rather a continuous concept that with every use, there is some level of disclosure/risk. There is no way to benefit from using data for social good/research with 0 risk. The more people understand this, the more constructive the conversations about the risk and benefits of using large datasets about people.
- A small paragraph on the pros/cons of approaches to privacy protection (e.g., simulated data, perturbed data, using secure data enclaves for access to real data) for ER research will be helpful to frame the paper and better understand when to use the simulated population or other alternatives. Some papers on perturbed data or using data enclaves are below.
  - Ramezani M, Ilangovan G, Kum HC. Evaluation of machine learning algorithms in a human-computer hybrid record linkage system. In CEUR workshop proceedings 2021 Jan (Vol. 2846, No. 4).
  - Ilangovan, Gurudev (2019). Benchmarking the Effectiveness and Efficiency of Machine Learning Algorithms for Record Linkage. Master's thesis, Texas A&M University. Available electronically from https : / /hdl .handle .net /1969 .1 /186390.
  - P. Christen, “Preparation of a real temporal voter data set for record linkage and duplicate detection research,” 2013.
  - Kum HC, Ahalt S. Privacy-by-design: Understanding data access models for secondary data. AMIA Summits on Translational Science Proceedings. 2013;2013:126.
- “We select a random 4% of birth events to be the birth of twins (two newborn simulants), and same last name, address, and date of birth.”: simulating multiple births is one of the key parts of making the simulated data useful for the intended purpose of ER. There is no reference to why 4%, it may be worth using a slightly higher percentage (see comment below). Also, twins often have even more similarity in my experience of real data (e.g., similar first name or same first letter in first name. SSN is only 1 digit off).  I am quoting from my published work in ER on this topic which includes a reference. “The most difficult links to resolve involve twins. In this case, much of the identifying information is validly the same or very similar. Often, SSNs are only one digit off, and one system might have assigned the SSN in one way, while another system has it assigned in the other way. These types of data errors make it almost impossible to automatically resolve entities without human intervention. Multiple birth rates have been rising in the United States, with twin birth rates at 29.3 per 1,000 births in 2000 [Reynolds et. al. 2003]. That is approximately six twins in every 103 children born, not including triplets and higher-order births. These are substantial numbers that need to be considered when performing record linkage on people level data. In one system of education data that performs record linkage on a regular basis, we have seen a twin field being regularly collected to differentiate data errors from real twins.” [Kum et. al. 2013]
  - Kum HC, Ahalt S, Pathak D. Privacy-preserving data integration using decoupled data. Springer New York; 2013.
  - Reynolds MA, Schieve LA, Martin JA, Jeng G, Macaluso M (2003) Trends in multiple births conceived using assisted reproductive technology, United States, 19972000. Pediatrics 111 (Supp 1):1159–1162
- Why is marriage or father not modeled in the births? Jr/Sr in the US naming culture do cause complexities in ER. So I am not sure I would agree with the stated hypothesis “We hypothesize that this limitation in realistic naming will not lead to substantially harder or easier data for linkage. “
- “In the event that model results did not meet verification and validation criteria, model implementation and/or design were iteratively adjusted appropriately until criteria were satisfied.”: it would be helpful to have some sense of how often these adjustments were needed, and how much of an adjustment was needed. This would help readers judge how close or not the final simulated results were before the fine tuning.
- “Thus, as time passes within the simulation, the proportion of households with irregular relationship structures grows.”: it is understandable that this is a limitation of this type of simulation, but a bit more explanation on issues such as the following would be useful.  (1) what are the issues related to this happening for ER; (2) how much is this a problem (e.g., rate of issues); and (3) limits on when the simulation should no longer be used due to this issue.
- “As with household physical and mailing addresses, when a business address is vacated, it is not reused. In effect, this likely makes business record linkage with these data easier than it will be in practice.” It is unclear why the business address is not reused especially given this statement that for ER for businesses it may not be a good choice since this was supposed to target ER usage.
- Also, how names for business were generated using graphs was confusing. Some concrete examples would be good to include.
- Given that this is based on the Vivarium simulation platform, some brief explanation of what it is would be helpful. Either not refer to it, if readers do not need anything about it or explain a little something about what it is beyond that it was used so that the paper can be somewhat stand alone.

[Clarification questions]

- “28-day time steps”: is there a reason not to use 30 days=1 month? 28 days seems to be the smallest days in a month, but unclear why this is good to do. It would seem that 1 year would not model well in this way. 30 days=360 days in a year. Which may be better.
- “When perturbation led to a negative age value, we flipped the negative age value’s sign”. Why not just use a perturbation that would not give a negative value? It seems that flipping the negative value would not result in the target distribution.
- “change employment randomly at a rate of 50 changes per 100 person-years (a rate we selected subjectively to provide an appropriate challenge in record linkage)”: It is unclear how employment is directly related to ER except maybe indirectly through change of address which in this particular simulation is tied to employment. Actually, it was unclear if employment change came before move, or the other way, or both. Also, unclear why this rate is appropriate. It maybe better to describe ER in terms of changes in address rather than employment. If not, a bit more clarity on how ER is related to employment would be helpful.
- “For instance, a child can move out of their household without a parent”: I assume this means even an infant can move out on their own. If we consider child adoptions/foster care this may be fine. But move out rates for children under 18, and adults should be quite different. This does not seem too difficult to implement and may be important to consider. Or for simplicity, maybe adoptions are not modeled but an age cutoff is implemented.

Are sufficient details of methods and materials provided to allow replication by others?

Partly

Is the rationale for creating the dataset(s) clearly described?

Yes

Are the datasets clearly presented in a useable and accessible format?

Partly

Are the protocols appropriate and is the work technically sound?

Partly

Reviewer Expertise:

Entity Resolution; Perturbing data for entity resolution; Privacy issues in and entity resolution

I confirm that I have read this submission and believe that I have an appropriate level of expertise to confirm that it is of an acceptable scientific standard, however I have significant reservations, as outlined above.

# Response

**Published:** 2024-10-02

**Authors:** Abraham Flaxman

**Affiliations:** University of Washington, Seattle, USA

**Comment: **This paper describes a python package that can simulate population data based on distributions of various individual and household characteristics in datasets released from the US census Bureau, with an explicit purpose for supporting and facilitating entity resolution (ER) algorithm and software development without accessing real confidential data. ER algorithms require access to personally identifiable information (PII) such as names, SSN, DOB making privacy a major concern in this type of research. Using simulated data, such as the one described in the paper is one approach to dealing with the privacy issues in ER. It relies on using the existing Vivarium simulation platform the same team developed, but attempts to incorporate some of the complexities of the ER problem with real data such as difficulties with twins.

 The paper is well written and easy to follow for the most part. Here are some questions/comments to consider for improvement.

***Response:** Thank you for this assessment, and for taking the time to offer this valuable feedback.*

**Comment: **[Major comments]

- Data errors (e.g., typos in names and dob, flipped first/last name in different systems) or variations (e.g., nicknames, change in last name due to marriage, Jr and Sr) are a common source of difficulties in ER using real data which does not seem to be modeled yet. Maybe at least mention it as important future work.

***Response: **Thank you for calling attention to this. We have developed the pseudopeople Python package (* *https://pseudopeople.org/* *) that includes additional affordances to add various kinds of noise to the simulated data described in this paper to afford a configurable amount of typos and other sources of error including some of those listed above, and we intend to add other noise such as the reviewer has identified in future updates to pseudopeople.  In this paper we hope to complement the pseudopeople documentation with a detailed description of the simulation process.*

*As we continue to add additional realism to challenge ER algorithms, it will be an interesting challenge to balance what we can make a configurable add-on (like the rate of first/last name flipping) and what we need to include in the simulation itself (e.g. the marriage-related name changes might need to be simulated together with the marriages) and therefore may not be able to make easily configurable.  We have added text to the abstract and introduction to emphasize that this paper is focused on the simulation and not the data errors.*

**Comment: **

- Similarly, duplicate records that exist in almost any real data make ER much more difficult because one cannot assume a one-to-one match unless the tables have been deduplicated first. One of the main difficulties in ER is that a duplicate record for one person cannot be mathematically differentiated from very similar records for 2 people such as twins, father/son, or even totally unrelated people (to date, I have met 2 people who told me after a ER talk that they know “of” this other person with the EXACT same first/last name and dob because they are in the same city and they have been confused before with the other person. Often it requires an explicit field stating that the person is a twin to be certain.

***Response: **As with our previous comment, this is an important area that we hope to address in more detail in future work, and we appreciate the reviewer calling attention to some of the unique challenges here. In our next round of pseudopeople enhancements, we plan to add “simple duplication” as an additional configurable type of “row_noise”. More complex duplication, such as may be caused by individuals who are reported in two distinct households during a decennial census, is something we have a start on in our simulation already and hope to also enhance in future work.  Another area for enhancement that we did not think of until reading this comment is the prospect of including an explicit field stating that the person is a twin --- just such a field is present in the Washington State Drivers License database (and was repurposed to create a twin registry for scientific research in Washington).*

**Comment: **

- I highly recommend reading the following paper for ideas for future work to make the simulation more realistic for ER research as well as adding a limitation section to the current paper. Such a section would be very important for potential users to know what the simulated data does not cover, and may need to still consider in their ER algorithm.
  - Goth G. Running on EMPI. Health information exchanges and the ONC keep trying to find the secret sauce of patient matching. Health data management. 2014;22(2):52-, 4, 6 passim.

***Response: **Thank you for pointing out this reference, and we agree that there are additional future work and limitations that it identifies admirable.  We believe that many (e.g. appending title to surname, as in FLAXMANPHD) which will be worthy additions to pseudopeople, but we did not feel any were so relevant to our simulation as to merit specific additional to the limitations section of this paper.*

**Comment: **

- Also, consider the following for the limitation section. It seems that this simulated data may be more supportive of ER algorithms that leverage structure (e.g., household structure or based on clustering) without a pair generation. However, many real world applications do not have much structure information to use, and may need to still consider pairwise ER algorithms. Complexities for this type of ER algorithms may not have been well modeled here (see point above).

***Response: **We appreciate the reviewer calling attention to the potential for using household structure in ER, which we agree is an underdeveloped avenue worthy of future research.  We agree that many real-world applications of ER will not be able to use such an approach, because it is not frequently available, and we hope that our sim and our python package will be a help in both developing novel methods and understanding when these methods are more or less applicable.*

**Comment: **

- A few sentences describing and talking about data governance/privacy concerns even for the simulated data in the introduction would be important. To my understanding, at a minimum a simulation of this scale may have simulatants that are too similar to real people, even if the data was all made up which may still give rise to privacy issues. It is very important that everyone becomes more educated on fundamental properties of information privacy. Such as, information privacy is not a binary property but rather a continuous concept that with every use, there is some level of disclosure/risk. There is no way to benefit from using data for social good/research with 0 risk. The more people understand this, the more constructive the conversations about the risk and benefits of using large datasets about people.

***Response: **Thank you for calling attention this important (and subtle) feature of simulated data of this scale. Both the generation and distribution of the dataset are governed by a system of “relational governance” which we developed for this simulated-but-realistic data together with the data ethicist Os Keyes. In Os’s approach, data subjects play a central role; a paper on their approach to data governance is currently under preparation. We have added a sentence to this effect to the introduction section.*

**Comment: **

- A small paragraph on the pros/cons of approaches to privacy protection (e.g., simulated data, perturbed data, using secure data enclaves for access to real data) for ER research will be helpful to frame the paper and better understand when to use the simulated population or other alternatives. Some papers on perturbed data or using data enclaves are below.
  - Ramezani M, Ilangovan G, Kum HC. Evaluation of machine learning algorithms in a human-computer hybrid record linkage system. In CEUR workshop proceedings 2021 Jan (Vol. 2846, No. 4).
  - Ilangovan, Gurudev (2019). Benchmarking the Effectiveness and Efficiency of Machine Learning Algorithms for Record Linkage. Master's thesis, Texas A&M University. Available electronically from https : / /hdl .handle .net /1969 .1 /186390.
  - P. Christen, “Preparation of a real temporal voter data set for record linkage and duplicate detection research,” 2013.
  - Kum HC, Ahalt S. Privacy-by-design: Understanding data access models for secondary data. AMIA Summits on Translational Science Proceedings. 2013;2013:126.

***Response: **We appreciate the reviewer’s suggestion and have added a sentence along these lines in the introduction. We will also consider all of these valuable references when framing and positioning our data governance approach in the paper that Os is currently working on.*

**Comment: **

- “We select a random 4% of birth events to be the birth of twins (two newborn simulants), and same last name, address, and date of birth.”: simulating multiple births is one of the key parts of making the simulated data useful for the intended purpose of ER. There is no reference to why 4%, it may be worth using a slightly higher percentage (see comment below). Also, twins often have even more similarity in my experience of real data (e.g., similar first name or same first letter in first name. SSN is only 1 digit off).  I am quoting from my published work in ER on this topic which includes a reference. “The most difficult links to resolve involve twins. In this case, much of the identifying information is validly the same or very similar. Often, SSNs are only one digit off, and one system might have assigned the SSN in one way, while another system has it assigned in the other way. These types of data errors make it almost impossible to automatically resolve entities without human intervention. Multiple birth rates have been rising in the United States, with twin birth rates at 29.3 per 1,000 births in 2000 [Reynolds et. al. 2003]. That is approximately six twins in every 103 children born, not including triplets and higher-order births. These are substantial numbers that need to be considered when performing record linkage on people level data. In one system of education data that performs record linkage on a regular basis, we have seen a twin field being regularly collected to differentiate data errors from real twins.” [Kum et. al. 2013]
  - Kum HC, Ahalt S, Pathak D. Privacy-preserving data integration using decoupled data. Springer New York; 2013.
  - Reynolds MA, Schieve LA, Martin JA, Jeng G, Macaluso M (2003) Trends in multiple births conceived using assisted reproductive technology, United States, 19972000. Pediatrics 111 (Supp 1):1159–1162

***Response: **Thank you for these valuable references. We use 4% of births without a suitable data source, and plan to enhance this in future work. Fortunately, our ad hoc number is similar to the evidence you shared --- our 4% of births for 2020-2040 is not too much higher than the 3% found in 2000. Your identification of similar SSNs is another challenge that we should incorporate when enhancing our twin generation component.*

**Comment: **

- Why is marriage or father not modeled in the births? Jr/Sr in the US naming culture do cause complexities in ER. So I am not sure I would agree with the stated hypothesis “We hypothesize that this limitation in realistic naming will not lead to substantially harder or easier data for linkage. “

***Response: **Good point, we did not consider the challenge of distinguishing between father and son with same names except for Jr and Sr suffixes.  We have removed this hypothesis, and in our next major update to our simulation, we hope to include fathers, as well as Jr/Sr naming practices.*

**Comment: **

- “In the event that model results did not meet verification and validation criteria, model implementation and/or design were iteratively adjusted appropriately until criteria were satisfied.”: it would be helpful to have some sense of how often these adjustments were needed, and how much of an adjustment was needed. This would help readers judge how close or not the final simulated results were before the fine tuning.

***Response: **This is a very reasonable request that is unfortunately very difficult to meet, due to a lack of careful information keeping.  We do have a complete revision history of the development of our model available publicly on GitHub, which (at the time I write this) shows* *640 code commits* *grouped into* *359 pull requests* *, but we do not have a straightforward way to identify which of these were changes in response to verification and validation issues. In the future, I think we can easily incorporate a “tagging” approach to PRs to identify code changes from V&V.*

**Comment: **

- “Thus, as time passes within the simulation, the proportion of households with irregular relationship structures grows.”: it is understandable that this is a limitation of this type of simulation, but a bit more explanation on issues such as the following would be useful.  (1) what are the issues related to this happening for ER; (2) how much is this a problem (e.g., rate of issues); and (3) limits on when the simulation should no longer be used due to this issue.

***Response: **We believe that this will not be an issue for most common approaches to ER, and will only be relevant for experimental approaches that use household structure as part of their linkage strategy. In the publicly available sample data, in the 2020 decennial census, 4% of rows have “Other nonrelative” as their relationship to the reference person.  In 2030, this rises to 16%, and in 2040, it is 20%. We plan to address this by adding more complex logic for relationship changes following migration in a future update. We have added a sentence about this to the limitations section.*

**Comment: **

- “As with household physical and mailing addresses, when a business address is vacated, it is not reused. In effect, this likely makes business record linkage with these data easier than it will be in practice.” It is unclear why the business address is not reused especially given this statement that for ER for businesses it may not be a good choice since this was supposed to target ER usage.

***Response: **This is a limitation that we plan to address in future update, and we agree with the reviewer that reusing business addresses will make this data more suitable for testing business linkage algorithms.*

**Comment: **

- Also, how names for business were generated using graphs was confusing. Some concrete examples would be good to include.

***Response: **Good idea, we have added a sentence with examples from the sample data distributed openly with the pseudopeople package.*

**Comment: **

- Given that this is based on the Vivarium simulation platform, some brief explanation of what it is would be helpful. Either not refer to it, if readers do not need anything about it or explain a little something about what it is beyond that it was used so that the paper can be somewhat stand alone.

***Response: **We feel that it is valuable to refer to the vivarium software that we used to implement this simulation and agree with the reviewer that it is important to include a brief explanation of what Vivarium is. Since we already attempted such an explanation in our original draft, we have revised it to include additional detail in the first paragraph of the methods section.  Perhaps the reviewer had some additional details in mind, however, and we welcome further feedback about how we can make this somewhat stand alone.*

 [Clarification questions]

**Comment: **

- “28-day time steps”: is there a reason not to use 30 days=1 month? 28 days seems to be the smallest days in a month, but unclear why this is good to do. It would seem that 1 year would not model well in this way. 30 days=360 days in a year. Which may be better.

***Response: **We prefer to work in units of days or weeks, since larger units of time like months and years have annoying variation in length.  So 28 days = 4 weeks is similar in length to one month, but always includes (for example) the same number of weekend days.  This is not particularly important for this simulation but can matter for simulations or data that included day-by-day variation.  We are confident that changing the timestep to 30 days would not lead to any difference in the difficulty of record linkage tasks with this simulated data.*

**Comment: **

- “When perturbation led to a negative age value, we flipped the negative age value’s sign”. Why not just use a perturbation that would not give a negative value? It seems that flipping the negative value would not result in the target distribution.

***Response: **We prefer this approach to obtaining a truncated distribution that does not include an unexpectedly high density at zero (as truncation would) and does not shift the mean as much as a non-negative perturbation (like adding an exponentially distributed offset) would. It is developed in detail in Bernard W. Silverman, Density Estimation for Statistics and Data Analysis, 1986 (p. 30).*

**Comment: **

- “change employment randomly at a rate of 50 changes per 100 person-years (a rate we selected subjectively to provide an appropriate challenge in record linkage)”: It is unclear how employment is directly related to ER except maybe indirectly through change of address which in this particular simulation is tied to employment. Actually, it was unclear if employment change came before move, or the other way, or both. Also, unclear why this rate is appropriate. It maybe better to describe ER in terms of changes in address rather than employment. If not, a bit more clarity on how ER is related to employment would be helpful.

***Response: **This particular ER challenge might be a bit niche, but it arises in systems like the US Census Bureau’s Person Identification Validation System where a tax id is available in employment records. As the reviewer notes, changes in address represent an important challenge in this setting, but changes in employment when address does not change could potentially be a barrier to high-quality record linkage in this setting as well.*

**Comment: **

- “For instance, a child can move out of their household without a parent”: I assume this means even an infant can move out on their own. If we consider child adoptions/foster care this may be fine. But move out rates for children under 18, and adults should be quite different. This does not seem too difficult to implement and may be important to consider. Or for simplicity, maybe adoptions are not modeled but an age cutoff is implemented.

***Response: **We do include some of this complexity in our current simulation, and as we write, “We modeled domestic migration events as happening at a rate determined by age, sex, and race/ethnicity”, but we also want to note that surprising or even illogical household composition can arise from the limited approach we have taken.  We hope to increase the realism of these dynamics in the future.*
